# Prognostic factors evaluated in differentiated thyroid carcinomas

**DOI:** 10.1530/EC-25-0751

**Published:** 2026-01-02

**Authors:** Yasuhiro Ito, Akira Miyauchi

**Affiliations:** Department of Surgery, Kuma Hospital, Kobe, Hyogo, Japan

**Keywords:** differentiated thyroid carcinoma, prognostic factors, papillary carcinoma, follicular carcinoma, static, dynamic

## Abstract

Differentiated thyroid carcinoma includes papillary (PTC) and follicular thyroid carcinomas (FTC). The biological characteristics and prognostic factors of these two carcinomas differ significantly. PTC is generally diagnosed by cytology; large tumor size, clinical node metastasis, and gross extrathyroidal and extranodal tumor invasion are significant prognostic factors for disease recurrence-free survival (DFS) and cause-specific survival (CSS). Age is a very important prognostic factor. Young age affects only DFS, whereas old age is related to both DFS and CSS. Aggressive variants, such as tall cell and hobnail variants, high Ki-67 labeling index, and *TERT* promoter gene mutations, are pathological and molecular prognostic factors. FTC is generally diagnosed based on postoperative pathology. Old age and large tumor size are associated with poor prognosis. Importantly, FTC is classified as minimally invasive, encapsulated angioinvasive, and widely invasive, which are closely related to the prognosis. Recent studies have demonstrated that the grade of vascular invasion is an important prognostic factor, whereas capsular invasion is not, because widely invasive FTC with no vascular invasion has an excellent prognosis. Dynamic markers, such as thyroglobulin doubling rate, metastatic tumor volume-doubling rate, and immunological indicators, such as the neutrophil-to-lymphocyte ratio, are useful for predicting the prognosis and evaluating the effectiveness of treatments, such as radioactive iodine therapy and molecular targeted therapy for recurrent lesions. Clinicians should accurately evaluate the prognostic markers of PTC and FTC in the pre-, intra-, and postoperative phases.

## Introduction

Differentiated thyroid carcinoma (DTC), the most common malignancy arising from thyroid follicular cells, comprises papillary (PTC) and follicular thyroid carcinomas (FTC) (1), which exhibit distinct biological characteristics. PTC is generally diagnosed via cytological examination, frequently metastasizes to regional lymph nodes, and extends to adjacent structures such as the recurrent laryngeal nerve, esophagus, and trachea. In contrast, FTC more frequently metastasizes to distant organs, such as the lung and bone, while lymph node metastasis and extension to adjacent structures are rare. As distinguishing between FTC and benign follicular adenoma using cytology alone is very difficult, FTC is typically diagnosed postoperatively by identifying capsular and/or vascular invasion on histopathological examination.

Owing to differences in their biological characteristics, the factors affecting patient prognosis differ between these carcinomas. Furthermore, prognosis should be evaluated at the three stages of carcinoma management: preoperative, intraoperative, and postoperative junctures (2). Preoperative prognostic factors are based on patient backgrounds and anatomical spread of the cancer evaluated with imaging findings, such as tumor size, presence or absence of clinical nodal metastasis (cN), and distant metastasis (cM). These factors are important in determining the surgical plan, including the extent of thyroidectomy, lymph node dissection, possible resection of adjacent organs, and the indication for postoperative radioactive iodine (RAI) therapy for ablation, adjuvant therapy, or distant metastasis. Intraoperative prognostic factors include surgical findings that affect the prognosis, primarily the invasion of adjacent organs by the carcinoma. Based on these findings, surgeons may change the extent of surgery, for example, converting a hemithyroidectomy to a total thyroidectomy and performing resection of the involved adjacent organs. Postoperative prognostic factors include static and dynamic factors. Representative statistical factors are determined based on pathological examinations and molecular testing; however, they predict patient prognosis only at the time of testing, not afterward. To date, some dynamic factors based on changes in blood markers such as thyroglobulin and/or metastatic tumor volumes have been identified, which are helpful for evaluating the disease condition of patients. These dynamic markers are useful for clinicians to determine whether and when to perform postoperative therapies, such as RAI and molecular-targeted therapies, and to evaluate their therapeutic effectiveness. In this review, both the static and dynamic prognostic factors for PTC and FTC are discussed.

In the past, the pathological distinction between PTC and FTC was not always clear. For example, follicular variant PTC, which was previously classified as FTC, has been reclassified as PTC, and the former noninvasive encapsulated follicular variant PTC has been separately classified from PTC and FTC as noninvasive follicular thyroid neoplasm with papillary-like nuclear features (1). It is therefore uncertain whether the previous classifications of PTC and FTC are relevant to the current classification system. Thus, in this review, we focused on recent studies reporting analyses of pathological specimens based on the current classification systems established over the past 10–15 years. Furthermore, in 2024, the English version of the clinical guidelines on the management of thyroid tumors conducted by the Japan Association of Endocrine Surgery was published (2). We referred to the risk classification of PTC and FTC based on these guidelines in this review.

## Static prognostic factors of PTC

### Factors based on preoperative findings

#### Age

Age is a well-established prognostic factor that, while inherently a continuous variable, is commonly analyzed using a defined age cutoff for convenience. According to the current tumor-node-metastasis (TNM) classification of the American Joint Committee on Cancer (AJCC)/Union for International Cancer Control (UICC), all patients without distant metastasis (M0) are classified as stage I if they are younger than 55 years (3). Previously, the cutoff age was 45 years; however, in 2010, Ito *et al.* reported that an age cutoff of 55 years more clearly reflected patient prognosis, including disease recurrence-free survival (DFS) and/or cause-specific survival (CSS) (4). After revision of the AJCC/UICC staging system, several studies have shown that 55 years is a suitable cutoff age for DFS and CSS (5, 6, 7). Nixon *et al.* and Mazurat *et al.* concluded that for disease-specific survival (DSS) in patients with DTC, an age cutoff of 55 years was more robust than 45 years (5, 6). Furthermore, Trimboli *et al.* showed that the application of an age cutoff of 55 years to the American Thyroid Association (ATA) stratification system could help identify patients with the highest risk of relapse for DFS (7). Some researchers have proposed different age cutoffs; for example, Sugitani *et al.* reported that 50 years was the optimal age cutoff for deriving DSS using their classification system (8). Sugino *et al.* showed that the DSS of patients with PTC without extrathyroidal extension was generally good regardless of age; however, receiver operating characteristic (ROC) curve analysis showed that the optimal cutoff age was 48 years for distinguishing patients with poor DSS (9).

Notably, the prognostic significance of age is not uniform in all patients with PTC. Ito *et al.* demonstrated that, in very low-risk PTC (T1aN0M0) according to risk classification based on the guidelines of the Japan Association of Endocrine Surgery (2), young patients (<55 years) were even more likely to show local recurrence than old patients (≥55 years). However, in low-risk patients (T1bN0M0), old patients showed a poorer distant DFS than young patients, and local DFS did not differ according to patient age (10). This may be because very low-risk PTC in young patients shows higher growth activity than that in older patients. For high-risk patients (PTC having tumor size >4 cm, cN ≥ 3 cm, extranodal tumor extension, extrathyroidal extension corresponding to T4a, or M1), old age significantly affected poor DFS and CSS (11). These findings indicate that although age is a very important prognostic factor, it is especially important in patients with PTC with high-risk features.

Some studies have proposed two cutoff ages for predicting patient prognosis: 40 and 60 years, 35 and 62.5 years, and 30 and 60 years (12, 13, 14). These studies showed that the prognostic significance of age in PTC is actually biphasic; that is, young patients are likely to experience recurrence, and old patients are likely to experience recurrence and die of thyroid carcinoma. Miyauchi *et al.* reported that patients aged <40 and >60 years showed a high proportion of biochemically persistent disease after total thyroidectomy, but only the latter group had a short thyroglobulin doubling time (Tg-DT) (12).

#### Male sex

The age, metastasis, extent, and size (AMES) classification sets different age cutoffs according to patient sex: 40 years for men and 50 years for women (15). To date, some studies have shown that male sex predicts a poor prognosis of PTC (15, 16, 17, 18, 19); however, this may be partially true because PTC in male patients shows more aggressive clinicopathological features, such as large size, multiple tumors, and positive node metastasis (15, 16).

In a single-institution study analyzing 5,897 patients with PTC, male sex was an independent prognostic factor for overall survival (OS) in patients aged <55 years (20), indicating that male sex affects PTC prognosis to some extent, although this was not adopted by the UICC/AJCC TNM classification.

#### Tumor size

Similar to age, this factor is a continuous function; however, for convenience, cutoffs are generally set to establish staging systems. The UICC/AJCC TNM classification specifies a 4 cm tumor size cutoff for upstaging patients aged ≥55 years (1), and the current ATA guidelines recommend lobectomy for low-risk DTCs measuring 1–4 cm (21, 22). The incidence of lobectomy increased from 13.7% (after the introduction of the 2009 guidelines) to 22.9% (after the introduction of the 2015 guidelines) (23). This cutoff is considered appropriate because previous clinical studies have demonstrated that a tumor size >4 cm significantly affected patients’ DFS, CSS, and OS (20, 24).

Previous studies have demonstrated that the prognostic value of conventional prognostic factors varies according to tumor size. Fukushima *et al.* reported that the prognostic significance of clinical lateral node metastasis (cN1b) was higher than that of extrathyroidal extension (T4a) in PTC ≤ 3.0 cm, whereas this significance was reversed in PTC > 3.0 cm (25). Ito *et al.* showed that extrathyroidal extension had a prognostic value for PTC > 2 cm, whereas cN affected prognosis even in papillary thyroid microcarcinoma (PTMC; T1aN0M0) (26). Moreover, Liu *et al.* reported a higher recurrence rate of macroscopic extrathyroidal extension in PTC > 1 cm, but no significant value in PTC ≤ 1 cm (27), which is consistent with the findings by Fukushima *et al.* (25).

#### Clinical lymph node metastasis (cN)

Numerous studies have been published on the prognostic significance of cN positivity; however, the characteristics of cN are also important. Researchers have investigated the prognostic significance of cN size by setting a cutoff value of 3 cm for convenience. In 2004, Sugitani *et al.* demonstrated that cN ≥ 3 cm was an important indicator of poor prognosis in patients aged ≥50 years with PTC (7). In high-risk PTC, cN ≥ 3 cm, age ≥55 years, tumor size >4 cm, and massive extrathyroidal extension were significantly associated with CSS (28). In 2010, Ito *et al.* classified the cN factor into three categories: cN0, no metastasis; cN1, metastasis ≤3 cm; and cN2, metastasis >3 cm or presence of extranodal tumor extension requiring at least partial excision of adjacent organs. Furthermore, they categorized PTC patients aged ≥55 years with cN1 as intermediate-risk and those with cN2 as high-risk. They proposed an intraoperative staging system (iStage) for patients ≥55 years: cN1 cT1-3 for iStage III, N2 any T, and cT4a any N for iStage IVA (29). Thereafter, they analyzed 5,768 patients with PTC and reported that cN ≥ 3 cm had the strongest prognostic impact on lymph node, lung, and bone recurrences (30). Therefore, in those aged ≥55 and <55 years, the investigators proposed the revision of the AJCC/UIOC staging system: upstaging of M0 PTC with cN ≥ 3 cm from stage II to stage III and from stage I to stage II, respectively (30). The risk classification system recommended by the Japan Association of Endocrine Surgery (JAES) classified patients with cN ≥ 3 cm and cN < 3 cm into high-risk and intermediate-risk categories, respectively (2). Another important cN characteristic is extranodal tumor extension, which is described in the ‘Factors based on intraoperative findings’ section below.

#### Distant metastasis at diagnosis (M1)

To date, numerous reports have been published on M1 as a DSS factor in FTC and PTC. However, the prognoses of patients with M1 vary according to clinicopathological features. One study on patients with M1 showed that large tumor size (>4 cm), old age (≥55 years), and T4a of the primary lesion independently predicted poor CSS (31), indicating that aggressive clinicopathological characteristics other than M1 and patients backgrounds affected CSS. Matsuzu *et al.* showed that age ≥55 years, metastasis to organs other than the lungs, and lack of avidity for RAI therapy were independent predictors of CSS in patients with M1 (32). Furthermore, Lee *et al.* showed that extensive extrathyroidal extension of the primary lesion resulted in poor outcomes in patients with PTC with initial distant metastasis (33). However, these studies have limitations because their analyses were based only on static markers. Analyses using dynamic markers are mandatory for patients having metastatic lesions, which are stated in the ‘Dynamic prognostic factors of PTC and FTC’ section below.

All these studies were conducted before the era of molecular targeted medicine. Recent and future developments may contribute to changes in the prognostic impact of distant metastasis or recurrence.

### Factors based on intraoperative findings

#### Extrathyroidal extension from primary lesions

Extrathyroidal extension is an important predictive factor of PTC prognosis. In the present version of the TNM classification (1), extrathyroidal extension is reflected in T factor levels and is classified as follows: T3b, gross extrathyroidal extension invading only strap muscles (e.g., extension to sternothyroid, sternohyoid, thyrohyoid, or omohyoid muscles); T4a, gross extrathyroidal extension invading subcutaneous soft tissues, larynx, trachea, esophagus, or recurrent laryngeal nerve; and T4b, gross extrathyroidal extension invading prevertebral fascia or encasing the carotid artery or mediastinal vessels. Curative surgery for T4b is often difficult, and even when it is implemented, Moritani *et al.* reported a dire prognosis of T4b patients aged ≥55 years (34).

T4a is the most important type of tumor invasion that significantly affects patient prognosis. Previous studies have demonstrated the prognostic value of T4a for patients with PTC (26, 29). However, the organs invaded by T4a PTC are diverse and complex. Thus, Ito *et al.* divided the T4a classification into two groups (30) (Table 1) according to the organ: invasion to shallow organs (T4a1) and deep organs (T4a2). They concluded that in the subset of M0 patients aged ≥55 years, the DSS rate of T4a2 patients was significantly poorer than that of T4a1 patients, and T4a1 patients showed similar DSS to stage II patients, indicating that it may be appropriate for T4a1 patients to be downstaged from stage III to stage II. Furthermore, among patients aged <55 years, the DSS rate of T4a2M0 patients was poorer than that of other stage I patients and did not differ from that of stage II patients (M1), indicating that T4a2M0 patients aged <55 years may be better upstaged to stage II.

**Table 1:** The subclassification of T4a proposed by Ito *et al.*

Classification	Organs invaded by PTC
T4a1	Tracheal adventitia and/or cartilage
(Shallow invasion)	Esophageal muscle layer
	Recurrent laryngeal nerve
	Cricothyroid and inferior pharyngeal constrictor muscles
T4a2	Subcutaneous soft tissues
(Deep invasion)	Larynx
	Tracheal mucosa
	Esophageal mucosa
	Jugular vein
	Brachiocephalic vein
	Sternocleidomastoid muscle

According to Ito *et al.* (30).

PTC, papillary thyroid carcinoma.

Controversial results have been reported regarding the prognostic value of T3b (35, 36, 37, 38), and Ito *et al.* investigated this issue by enrolling 7,811 patients with M0 PTC with long-term follow-up (median 10.0 years) (35). In the subset of ≥55 years, the lymph node recurrence-free survival (LR-FS), distant recurrence-free survival (DR-FS), and CSS of T3bN0M0 patients were significantly poorer than those of stage I patients and did not differ from those of T3aN0M0 patients. Furthermore, the CSS of T3aM0 patients did not differ from that of T4a1N0 patients. Therefore, in patients aged ≥55 years, it is appropriate that T3bM0 patients are set at stage II.

#### Extranodal tumor extension (LNEx)

PTC invasion of the surrounding tissues occurs not only in primary lesions but also in metastatic lymph nodes. In 2014, Moritani *et al.* reported that LNEx was related to poor prognosis, although it was not an independent prognostic factor in multivariate analysis (39). In 2007, Ito *et al.* showed that LNEx significantly affected DFS and DSS in both univariate and multivariate analyses (40), and in 2018, they demonstrated that LNEx was an independent prognostic factor for OS (20). Thereafter, studies published in other countries reported the prognostic significance of LNEx in PTC (41, 42, 43, 44, 45, 46, 47, 48). Suh *et al.* performed a meta-analysis showing that LNEx is a marker of poor prognosis (43), and Kim *et al.* claimed that the incorporation of LNEx in the ATA classification improves the accuracy of risk stratification for patients with thyroid carcinoma (44). A few recent studies have also reported the prognostic value of LNEx (47, 48, 49), and Ito *et al.* showed that patients with LNEx positivity are appropriate for upstaging to stage II if they are <55 years and to stage III if they are ≥55 years of age (49). In the risk classification of PTC conducted by the JAES, LNEx was adopted as a high-risk feature (9).

In contrast to extrathyroidal extension, no studies have examined differences in prognosis based on the organs invaded by the metastatic nodes. This may be because the incidence of LNEx is much lower than that of extrathyroidal extension. However, future studies are needed to more accurately predict patients’ prognoses.

The TNM classification is simple, clear, and easy to use in various institutions worldwide. It expresses the extent of the anatomical spread of the cancer. Ex, N, and M represent local invasiveness, nodal metastasis, and distant metastasis, respectively. However, this system is limited because it does not consider the growth activity of cancer.

### Factors based on postoperative findings

#### Aggressive pathological variants

In addition to the conventional subtype, PTC has several other pathological subtypes. The prognosis of PTC is not uniform regardless of subtype, and some aggressive subtypes have been identified, such as tall cell, columnar cell, and hobnail variants (50, 51, 52). Furthermore, in the latest WHO classification, a new disease classification has been established, namely high-grade follicular cell-derived nonanaplastic thyroid carcinoma, which is subdivided into poorly DTC and differentiated high-grade thyroid carcinoma (53). Although the incidence of these malignancies is low (up to 6.7%), the prognosis of patients with these diseases is generally poor (54). Pathologists should diagnose tumors not only as PTC but also as aggressive variants, and clinicians should treat patients diagnosed with such variants carefully, even if no other high-risk features can be detected.

#### Cell proliferating activity (Ki-67 labeling index (LI))

Ki-67 is a cell proliferation-associated antigen expressed at all stages of cell proliferation except in the G0 phase. The expression of Ki-67 is evaluated immunohistochemically as a Ki-67 LI in tissue specimens. The evaluation of Ki-67 LI is simple and useful for evaluating PTC growth and prognosis. It has been identified as a prognostic factor for both DFS and CSS in patients with PTC (55, 56, 57, 58, 59, 60, 61). Some studies reported the prognostic value of Ki-67 LI in combination with other factors (56, 57). According to Matsuse *et al.*, the combination of high Ki-67 LI and *TERT* promoter gene mutations is strongly associated with DFS in patients with PTC (56). Miyauchi *et al.* showed that Ki-67 LI was significantly associated with biochemically persistent disease and was inversely related to Tg-DT (see the ‘Dynamic factors’ section) (57). The authors concluded that the evaluation of Ki-67 LI in primary tumors may allow prediction of postoperative Tg status, Tg-DT, and prognosis of patients with PTC.

#### *TERT* promoter mutations

In 2014, Xing *et al.* reported that the association between *BRAF* V600E and *TERT* promoter mutations was a predictor of PTC recurrence (62, 63). However, they demonstrated that the prognosis of PTC positive for *TERT* promoter mutations but negative for *BRAF* V600E mutations was not poor. In 2020, Ebina *et al.* showed that patients with PTC with *TERT* promoter mutations had poorer CSS than those without mutations (10-year CSS rates 73.7% vs 98.1% and 10-year DFS rates 53.7% vs 93.3%) (43). They did not investigate the *BRAF* V600E mutation; therefore, their series included cases lacking the *BRAF* V600E mutation, although the number was small. As reported by Xing *et al.* (62), the incidence of such tumors should be low and should not significantly affect their analysis. As the prognosis of patients with intrathyroidal PTC measuring 1.1–4 cm did not depend on the extent of thyroidectomy (total thyroidectomy or lobectomy), the authors speculated that total thyroidectomy is overtreatment for such PTCs. Additional studies have been published on *TERT* promoter mutations and prognosis (53, 64, 65, 66).

### Summary

Table 2 summarizes the static prognostic factors for PTC based on pre-, intra-, and postoperative findings. These factors significantly influence therapeutic strategies at each point. Pre- and intraoperative evaluations are crucial for determining the surgical plan (extent of thyroidectomy and prophylactic/therapeutic lymph node dissection) and whether postoperative adjuvant therapy, such as RAI administration, should be performed. Postoperative evaluation is important for attending clinicians when determining appropriate follow-up strategies, especially in situations where careful monitoring is required and referral to a clinic in the patient’s hometown is not feasible. For high-risk cases, more intensive surveillance – including measurement of Tg and Tg-Ab levels, ultrasonography, and other imaging studies such as computed tomography (CT) and positron emission tomography (PET)-CT – may be necessary.

**Table 2:** Summary of static prognostic factors for PTC based on pre-, intra-, and postoperative findings.

	Moderate	Strong
Preoperative findings	Young age (DFS only) (<30 years) Male sex Tumor size >4 cm (T3a)	Old age (≥55 or ≥60 years) (especially for cases with other risk factors) M1
Intraoperative findings	*n* < 3 cm	*n* ≥ 3 cm
	Ex corresponding to T3a and T4a1*	Ex corresponding to T4a2* and T4b
Postoperative findings		High Ki-67 LI (5% or 10%)
		Aggressive subtypes
		*TERT* promoter gene mutations

PTC, papillary thyroid carcinoma; DFS, disease recurrence-free survival; DSS, disease-specific survival; *n*, clinical lymph node metastasis; Ex, extrathyroidal extension; LNEx, extranodal tumor extension; LI, labeling index.

* See the ‘Extrathyroidal extension from primary lesions’ section and Table 1.

## Static prognostic factors of FTC

As described in the ‘Introduction’ section, FTC is generally diagnosed via postoperative pathological examination, except in the presence of distant metastases. The prognosis of FTC is most accurately predicted based on postoperative pathological examination.

### Factors based on preoperative findings

#### Age

Similar to PTC, age is an important prognostic factor in FTC. A meta-analysis showed that age >45 years is a prominent prognostic factor for DSS (67). Ito *et al.* divided patient age into three categories: <20, 20–44, and ≥45 years. In the subsets of both minimally and widely invasive FTC, DFS and DSS were poorest in patients aged ≥45 years, and the DFS of patients aged <20 years was poorer than that of patients aged 20–44 years (68). Furthermore, the superiority of a cutoff age of 55 years compared with a cutoff age of 45 years was reported (69), and Ito *et al.* showed that age ≥55 years was an independent predictor of distant recurrence (70). Therefore, it can be concluded that the prognostic value of age in FTC has a similar trend to that in PTC; young patients are likely to show recurrence, and older patients are likely to show recurrence and death from thyroid carcinoma. In 2001, Mazzaferri and Kloos reported high incidences of local and distant recurrences and low disease-specific mortality in young patients with PTC and FTC, whereas older patients had high incidences of recurrence and high disease-specific mortality (71). The latter is easy to understand, whereas the former is more difficult to explain in young patients. A possible reason for this is that PTC and FTC in young patients are more responsive to radioactive iodine and thyroid-stimulating hormone suppressive management with levothyroxine than in older patients. Miyauchi discussed this phenomenon and explained the little-known spontaneous regression of thyroid cancers (72).

#### Others

M1 has also been identified as a DSS factor. Furthermore, a tumor size >4 cm and male sex have been reported to have prognostic values, although not strong (67, 73).

### Factors based on postoperative findings

#### Vascular and capsular invasion

According to the latest WHO classification, FTC is divided into three categories based on capsular invasion (CI) and vascular invasion (VI). Minimally invasive FTC has microscopically minimal CI but no VI, encapsulated angioinvasive FTC has VI with no or minimal CI, and widely invasive FTC has a wide CI (1). However, there are a few important issues associated with the classification of FTC. First, an accurate definition of widespread CI is lacking. In hospitals specializing in thyroid disease, widely invasive FTC is diagnosed if CI is grossly detected on the incised surface; however, the definition of wide CI has not been unified worldwide. Second, a description of whether VI is associated with wide CI in widely invasive FTC is lacking. Therefore, it remains unclear whether VI is mandatory for the diagnosis of widely invasive FTC. This issue is important because studies from two Japanese institutions have demonstrated that widely invasive FTC with VI(−) shows excellent prognoses (72, 74, 75). Ito *et al.* showed that the DR-FS rates between minimally invasive FTC and VI(−) widely invasive FTC did not significantly differ, whereas Vi(+) widely invasive FTC showed a very poor prognosis (70).

FTC is diagnosed as angioinvasive if one or more VI lesions are detected. However, Ito *et al.* demonstrated that angioinvasive FTC with ≥4 VI showed a significantly poorer DFS than that with <4 VI (70). Therefore, they suggested that subclassification is necessary to more accurately reflect patient prognosis according to the number of VI. Yamazaki *et al.* showed that angioinvasive FTC VI ≥ 2 showed a poorer prognosis than FTC with VI = 1 (74).

Based on these findings, Table 3 presents the classification of FTC according to patient prognosis. These classifications were used for M0 patients. However, since M1 FTCs show a poor prognosis regardless of the pathological type of the primary lesion (70), M1 FTC should be classified as having a poor prognosis.

**Table 3:** Classification of FTC based on pathological findings according to patient prognosis.

Prognosis	Classification of FTC
Excellent	Minimally invasive
	Widely invasive (VI-negative)
Moderate	Encapsulated angioinvasive (*small number of VI lesions)
Poor	Widely invasive (VI-positive)
	Encapsulated angioinvasive (^†^large number of VI lesions)

* <4 lesions.

^†^ ≥4 lesions

FTC, follicular thyroid carcinoma; VI, vascular invasion.

#### Ki-67 LI

Ito *et al.* demonstrated that a high Ki-67 LI (≥5%) has a significant prognostic value for DFS in both minimally invasive and widely invasive FTC (based on the former WHO classification) (76, 77). Hellgren *et al.* demonstrated that Ki-67 LI in FTC was higher than that in benign adenoma; Ki-67 LI was an independent prognostic factor with an optimal Ki-67 LI cutoff of 4% by ROC curve (78).

### Summary

Pathological results are important for predicting the prognosis of FTC. However, many studies on FTC prognosis were published before the establishment of the present pathological classification: minimally invasive, widely invasive, and angioinvasive. Recent studies analyzing these three types of FTC have shown that in M0 tumors, i) minimally invasive FTC and widely invasive FTC with VI(−) have an excellent prognosis, ii) widely invasive FTC with VI(+) shows a poor prognosis, and iii) the prognosis of angioinvasive FTC depends on the number of VI foci.

Since most patients with suspected FTC initially undergo hemithyroidectomy, completion of total thyroidectomy is recommended for patients with a poor prognosis. Thus, patients diagnosed with widely invasive FTC with VI positivity or angioinvasive FTC with extensive invasion should undergo completion total thyroidectomy for accurate monitoring of serum Tg levels and preparation for distant recurrence requiring RAI therapy. The combination of high Ki-67 LI, old age, and male sex is helpful in determining completion of total thyroidectomy.

## Dynamic prognostic factors and immunological indicators of PTC and FTC

### Dynamic prognostic factors

To date, several dynamic prognostic factors have been identified for PTC and FTC. The values of these factors change over time, and evaluation of these changes is useful for predicting patient outcomes.

#### Thyroglobulin doubling rate (Tg-DR)/thyroglobulin-doubling time (Tg-DT)

Tg-DR/Tg-DT was proposed by Miyauchi in 2011, which accurately reflects patient prognosis in real time (79). Short Tg-DT was significantly related to DSS in patients with PTC who underwent total thyroidectomy and were negative for Tg-Ab. The calculation formula is complicated; however, it can be downloaded from the homepage of Kuma Hospital (https://en.kuma-h.or.jp/tools).

However, DT has two limitations. First, in patients showing a decrease in Tg levels or tumor volumes over time, the DTs become negative (<0). When these are analyzed with DT-positive cases, there is a discontinuity problem between the DT-positive and DT-negative values. Second, the DT values are opposite to the tumor growth rate values. These limitations were solved by adopting an inverse DT value (1/DT), named the doubling rate (DR), by Miyauchi *et al.* (78, 80). The DR indicates the number of doubling or halving events that occur per unit time.

#### Tumor volume doubling rate (TV-DR)/tumor volume-doubling time (TV-DT)

Changes in tumor volume (TV) have a prognostic impact that can be evaluated using DR/DT. The maximum diameter (D1) and the diameter in the direction perpendicular to the maximum diameter (D2) are measured, and the tumor volume is calculated using the formula (π/6 × D1 × D2 × D2). The calculation software can be downloaded as described above. Sabra *et al.* showed that TV-DT of distant metastatic lesions was significantly related to DSS in patients with DTC (81). In 2021, Ito *et al.* showed that high TV-DR (>1/year), together with Tg-DR >1/year, was an independent predictor of CSS in patients having DTC with distant metastasis on multivariate analysis with conventional prognostic factors (82).

### Neutrophil-to-lymphocyte ratio (NLR) as an immunological indicator

The NLR is an immunological marker that reflects the imbalance between immune surveillance and tumor progression; an increased number of neutrophils and a decreased lymphocyte ratio are thought to reflect carcinoma progression and impaired immunological surveillance, respectively. The NLR can be used both for initial surveillance before surgery and for monitoring changes in its value over time during follow-up. This marker has been reported to have a strong prognostic impact on various carcinomas; however, no prominent findings have been detected (83, 84, 85, 86, 87, 88), although one study showed a relationship between changes in NLR (NLR at 6–18 months after surgery minus NLR before initial surgery) and incomplete response to therapy (89). Ito *et al.* focused on the NLR for patients with DTC having distant metastasis and reported that an NLR > 3 at the first detection of distant metastasis was an independent predictor of carcinoma death in patients (82). Furthermore, after the NLR reached >3, the CSS was very poor, with 5-year and 10-year CSS rates of 50.4 and 23.9%, respectively. Therefore, NLR can be a useful marker for the progression of Tg-Ab-positive DTC. The relationship between the increasing rate of the NLR and patient prognosis warrants further research.

In addition, NLR may be useful for evaluating the effects of postoperative therapies for metastatic lesions, such as molecular-targeted medicine. Ito *et al.* showed that changes in not only the Tg levels but also the NLRs of patients with DTC who underwent sorafenib or lenvatinib treatment strongly reflect therapeutic efficacy (90). Fukuda *et al.* showed that patients with DTC with a median NLR <3 at the initiation of lenvatinib therapy had a longer OS and that the median NLR values decreased when the best tumor response was achieved, whereas they increased again with disease progression (91).

### Summary

Dynamic factors and immunological indicators are useful for estimating the progression of recurrent lesions during postoperative follow-up and for determining whether treatment intervention is required. Both Tg-DR and TV-DR significantly reflected disease progression and CSS. Further studies on NLR are needed, particularly on changes in NLR levels, as it may have prognostic value, especially in Tg-Ab-positive patients.

## Summary and future prospects

In this review, we discussed the pre-, intra-, and postoperative prognostic factors for PTC and FTC. Pre- and intraoperative factors are useful for planning the surgical design and postoperative management, including adjuvant treatment. Postoperative findings should be carefully evaluated to avoid oversight of important pathological and molecular findings. During postoperative follow-up, clinicians should consider changing dynamic factors and immunological indicators to appropriately implement adjuvant/therapeutic RAI administration and, if needed, molecular-targeted therapy.

The most recently published ATA guidelines also include risk classification for PTC, FTC, and oncocytic thyroid carcinoma (22). There are some discrepancies in high-risk features between the ATA guidelines and our classification system (2). The ATA guidelines classify T3b tumors as high-risk, although our study showed differing findings (35). They also classify all widely invasive FTC as high-risk, an upstaging from the 2016 guidelines (21), whereas we demonstrated that VI is an important factor for predicting prognosis in widely invasive FTC (70). Furthermore, R1 (microscopically incomplete resection) was downstaged to low-intermediate risk in the newest guidelines (22). This factor is difficult to analyze, and we have no data on this issue. Further studies with larger patient cohorts are necessary to elucidate all these points.

How to include the size of the primary tumor, metastatic nodes, and patient age in the risk classification remains an open question. Generally, these are included by setting one or more cutoff values. However, in contrast to other factors, tumor size and age are fundamentally continuous. Therefore, it is strange that patient prognoses change significantly and suddenly at certain points in age and size, such as 55 years and 4.0 cm, respectively. In 1993, Hay *et al.* established their own prognostic scoring system, with age and tumor size as continuous variables (92), which was designated as MACIS (metastasis, age, completeness of resection, invasion, and size). Recently, some more important factors, such as N status and subclassification of extrathyroidal and extranodal invasions, have been identified, which should be added to such a calculation system. We expect that a novel scoring system will be established in the future for more accurate evaluation of DTC prognosis in patients by including age and tumor size as continuous variables.

## Declaration of interest

The authors declare that there are no conflicts of interest that could be perceived as prejudicing the impartiality of this work.

## Funding

This work did not receive any specific grants from funding agencies in the public, commercial, or not-for-profit sectors.
